# Interaction Induced High Catalytic Activities of CoO Nanoparticles Grown on Nitrogen-Doped Hollow Graphene Microspheres for Oxygen Reduction and Evolution Reactions

**DOI:** 10.1038/srep27081

**Published:** 2016-06-03

**Authors:** Zhong-Jie Jiang, Zhongqing Jiang

**Affiliations:** 1 New Energy Research Institute, College of Environment and Energy, South China University of Technology, Guangzhou 510006, Guangdong, China; 2 Department of Chemical Engineering, Ningbo University of Technology, Ningbo 315016, Zhejiang, China

## Abstract

Nitrogen doped graphene hollow microspheres (NGHSs) have been used as the supports for the growth of the CoO nanoparticles. The nitrogen doped structure favors the nucleation and growth of the CoO nanoparticles and the CoO nanoparticles are mostly anchored on the quaternary nitrogen doped sites of the NGHSs with good monodispersity since the higher electron density of the quaternary nitrogen favors the nucleation and growth of the CoO nanoparticles through its coordination and electrostatic interactions with the Co^2+^ ions. The resulting NGHSs supported CoO nanoparticles (CoO/NGHSs) are highly active for the oxygen reduction reaction (ORR) with activity and stability higher than the Pt/C and for the oxygen evolution reaction (OER) with activity and stability comparable to the most efficient catalysts reported to date. This indicates that the CoO/NGHSs could be used as efficient bi-functional catalysts for ORR and OER. Systematic analysis shows that the superior catalytic activities of the CoO/NGHSs for ORR and OER mainly originate from the nitrogen doped structure of the NGHSs, the small size of the CoO nanoparticles, the higher specific and electroactive surface area of the CoO/NGHSs, the good electric conductivity of the CoO/NGHSs, the strong interaction between the CoO nanoparticles and the NGHSs, etc.

Unitized regenerative fuel cells (UNFCs) and metal-air batteries have been identified as the promising energy conversion and storage devices with great potential to alleviate the ever-increasing pressure from the energy deficiency^1^,^2^,^3^,^4^. However, their widespread applications in the real-world devices have been greatly hindered by the sluggish kinetics of oxygen reduction reaction (ORR) and oxygen evolution reaction (OER), both of which are the key electrode processes of UNFCs and metal-air batteries^3^,^4^,^5^,^6^. The development of oxygen electrode catalysts that are active for both the ORR and the OER presents a great challenge, since the catalysts efficient for the ORR usually tend to be poor for the OER and vice versa^7^,^8^. For instance, precious metals such as platinum (Pt) and its alloys which are well-known ORR catalysts are poor for the OER, while iridium and ruthenium oxide-based catalysts which have extraordinary OER activity exhibit low ORR activity^9^,^10^,^11^,^12^. Although integration of precious metals with iridium/ruthenium oxide might be a promising way to fabricate bifunctional catalysts with high efficiency for the ORR and the OER^13^,^14^,^15^, their practical uses in UNFCs and metal-air batteries have been greatly limited by high cost, scarcity, and poor stability. Extensive efforts have therefore been devoted to the development of alternative low-cost and high-performance oxygen electrode catalysts with improved efficiency and stability^8^,^16^,^17^.

Recent work has demonstrated the potential use of transition-metal oxides (TMOs) as bifunctional catalysts for the ORR and the OER^1^,^8^,^18^,^19^,^20^. However, the TMOs prepared by the traditional synthetic routes usually possess large particle sizes and low specific surface areas, which, along with the low electric conductivity^21^,^22^, have greatly reduced the electrocatalytic activities of the TMOs for the ORR and the OER. Strategies employed to improve the electrocatalytic activities of the TMOs include reducing the particles to nanometer size scale, doping them with electron donor and integrating them with conducting materials^23^,^24^,^25^.

Graphene, a layer of sp^2^ bonded carbon atoms arranged in a honeycomb (hexagonal) lattice, is a promising conducting material due to its superior electric conductivity^26^,^27^. However, simple physical mixing of graphene with the TMOs would invariably lead to high interfacial electron transfer resistance, which might not be desirable to improve the electrocatalytic activities of the TMOs^28^,^29^. Although reduction of the interfacial electron transfer resistance could be achieved by intimate contact of graphene with TMOs through direct nucleation and growth of the TMOs on the graphene nanosheets, the development of such hybrid materials has indeed been hindered by the chemical inertness of graphene. Specific surface modification of graphene is therefore required to create suitable chemical environments for nucleation and growth of the TMOs^24^,^30^. The doping with nitrogen is an alternative way to make graphene available for the deposition of the TMOs, since nitrogen containing groups in nitrogen doped graphene (NG) with high electron density could act as the centers coordinating with transition metal ions in the reaction mixture, facilitating the nucleation of the TMOs and the subsequent growth of the TMO particles with small sizes. In such NG supported TMOs (TMOs/NG), the improved electric conductivity of NG due to its nitrogen doped structure could make an additional contribution on improving the electrocatalytic activities of the TMOs. It is recently demonstrated that NG is also electrocatalytically active for both the ORR and the OER^31^,^32^,^33^, which makes the TMOs/NG even more attractive as the bifunctional catalysts, since collective interactions between NG and TMOs may enhance their functionalities for the catalytic ORR and OER.

Although the integration of NG shows great potentials to increase electrocatalytic activities of the TMOs, its practical uses might be hampered by the extremely large aspect ratio of the NG nanosheets. As reported previously, an intimate van der Waals interaction between the NG nanosheets might lead to an embedment of the TMO particles in the interlayer spacing of the NG nanosheets^21^,^34^,^35^, which would pose a great constraint on the accessibility of the TMOs to the electrolyte due to isolation of the TMO particles from the electrolyte. An appealing strategy to avoid the embedment of the TMOs in the interlayer spacing of the NG nanosheets is to directly deposit them into a 3-dimensional porous NG material. Porous materials could not only allow the accessibility of the electrolytes to their surfaces, but throughout the bulk of the materials, which is of great significance to improve the utilization of the catalysts and thereby their catalytic activities. As demonstrated by some recently published work, the TMO particles deposited in the 3-dimensional graphene based materials could exhibit extremely higher electrochemical performance as the electrode materials for lithium ion batteries and quasi-supercapacitors, since the porous morphologies allow for the better diffusion of the electrolytes and higher utilization of the active materials^36^,^37^,^38^.

In this work, we would employ nitrogen doped graphene hollow microspheres (NGHSs) as the supports for the deposition of CoO, a typical TMO which is electrocatalytically active for the ORR and the OER. It shows that the nitrogen doped structure could not only facilitate the nucleation and growth of the CoO nanoparticles on the surface of the NGHSs, but also promote the interaction between CoO and NGHSs. The CoO nanoparticles are mainly anchored on the quaternary-type nitrogen, leading to a strong interaction between CoO and NGHSs. The random stacking of these NGHSs supported CoO nanoparticles (CoO/NGHSs) would lead to the formation of a solid with a 3-dimensional porous structure. The CoO/NGHSs are highly active for the oxygen reduction reaction (ORR) with activity and stability higher than the commercial Pt/C 20 wt.% and for the oxygen evolution reaction (OER) with activity and stability comparable to the commercial RuO_2_/C 20 wt.% and the most efficient catalysts reported to date, suggesting a great potential of using the CoO/NGHSs as bifunctional catalysts for the ORR and the OER.

## Results and Discussions

**Figure 1:**
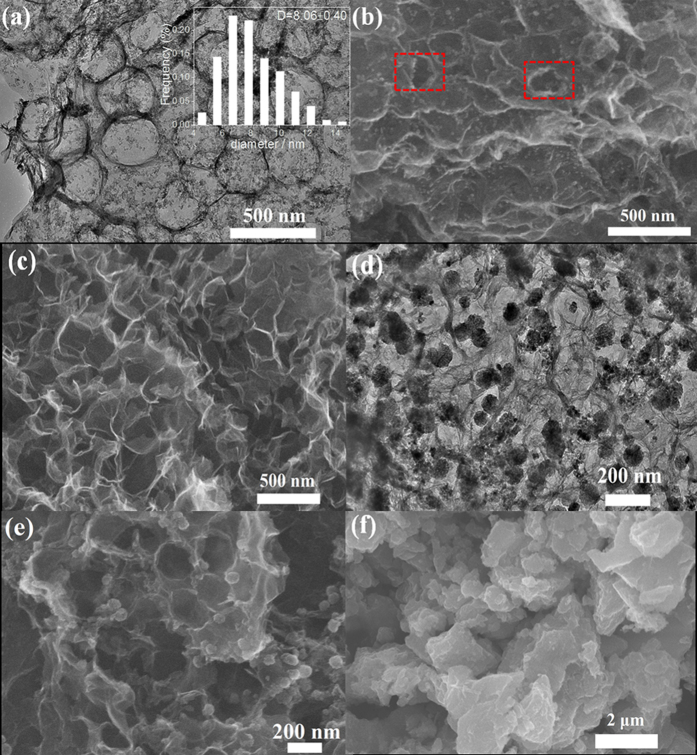
TEM (**a**) and SEM (**b**) images of the CoO/NGHSs; (**c**) SEM image of the NGHSs; (**d**) TEM image of the CoO/GHSs; (**e**,**f**) SEM images of the CoO/GHSs and the pure CoO solid grown in the absence of the substrate. The red boxes in the Fig. 1b show the damaged NGHSs.

The CoO/NGHSs reported in this work were synthesized from a procedure involving the preparation of the positively charged polystyrene (PS) spheres (the ζ-potential of the PS spheres is 51.6 ± 0.25 mV at pH 5.0–7.0, see the supporting information), the fabrication of the GO/PS composites by adsorption of the negatively charged GO onto the surface of the PS spheres through the electrostatic interaction (the SEM image of the GO/PS spheres shown in the Fig. S1a), the formation of the NGHSs through the calcination of the GO/PS composites in the presence of melamine, and the subsequent deposition of the CoO nanoparticles. Figure 1a shows a typical TEM image of the obtained CoO/NGHSs, in which the hollow structure of the NGHSs can be clearly identified by their pancake-like morphology with the opaque peripheries. TEM also shows the presence of some sheet-like materials (Fig. 1a), which could be attributed to free nitrogen doped graphene (NG) formed from GO unadsorbed on the PS spheres during the fabrication of the GO/PS composites, or NG formed from the damaged NGHSs, since the ultrasonication and mechanical stirring during the washing and sample preparation for TEM imaging may destroy the specific hollow structure of the NGHSs. The damage of the hollow structure of some NGHSs could also be observed by their SEM image shown in Fig. 1b (as marked with red boxes). The absence of the well-defined spherical morphology of the NGHSs in the SEM image of the CoO/NGHSs (Fig. 1b) indicates the squash of the NGHSs by the CoO nanoparticles due to their highly pliable nature of the graphitic structure and specific hollow morphology, which cannot well support the gravity of the CoO nanoparticles. This is unlike the SEM image of the pure NGHSs shown in Fig. 1c, where the spherical morphology of the NGHSs could be clearly visualized due to the absence of the CoO nanoparticles. Figure 1a shows that the CoO nanoparticles on the surface of the NGHSs are well separated and monodispersed with an average size of 8.06 ± 0.40 nm, as shown by the histogram in the inset of Fig. 1a. This is consistent with the higher-magnification TEM image of the CoO/NGHSs in Fig. S2a, where the CoO nanoparticles with the average size of ∼8 nm on the NGHSs could be clearly observed. The high-resolution TEM image of the CoO/NGHSs in Fig. S2b shows that the CoO nanoparticles are well crystallized. Based on the lattice fringes shown in Fig. S2b, it can be inferred that the CoO nanoparticles have a rock salt cubic CoO structure. Control experiments show that the growth of the CoO particles in the absence of the supports or in the presence of the GHSs (the SEM image of the GHSs shown in the Fig. S1b) would lead to the formation of the CoO solid with irregular shapes or the CoO aggregates consisting of the CoO sub-nanoparticles with relatively larger sizes, as shown in Fig. 1d–f. This indicates that the presence of the nitrogen doped structure is crucial to obtain the CoO nanoparticles on the NGHSs with small sizes and good monodispersity. These nitrogen doped sites may act as the anchoring points for the nucleation and subsequent growth of the CoO nanoparticles due to the strong interaction between Co^2+^ ions and N atoms.

**Figure 2:**
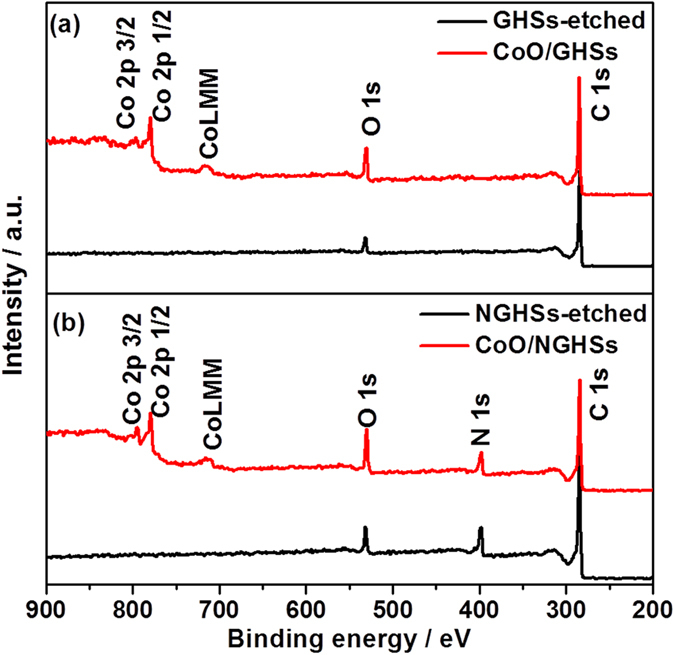
(**a**) XPS survey spectra of the CoO/GHSs and the GHSs-etched; (**b**) XPS survey spectra of the CoO/NGHSs and the NGHSs-etched.

**Figure 3:**
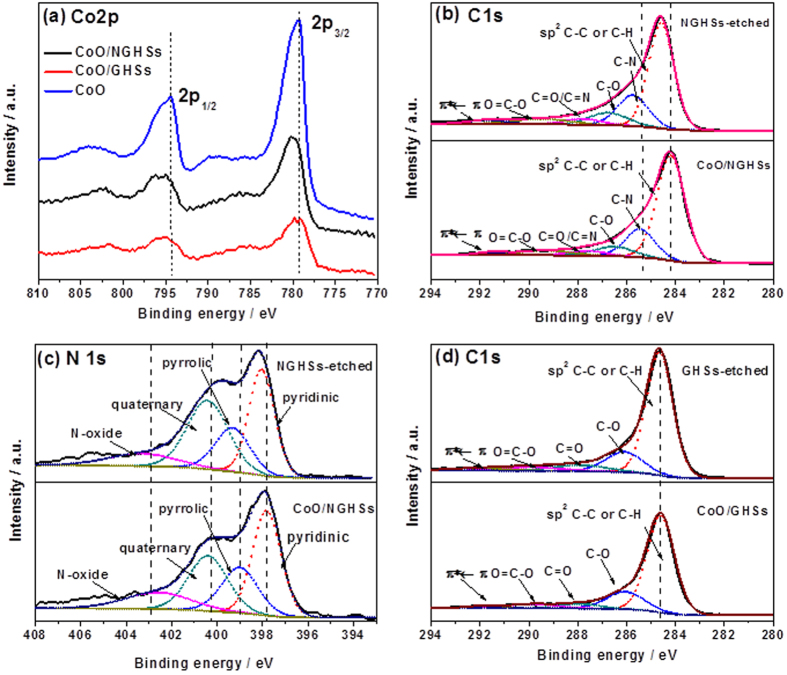
(**a**) High-resolution XPS Co 2p spectra of the CoO/NGHSs, the CoO/GHSs, and the pure CoO solid; (**b**) Deconvoluted XPS C 1s spectra and (**c**) deconvolution of the XPS N 1s spectra of the CoO/NHGSs and the NHGSs-etched; (**d**) Deconvoluted XPS C 1s spectra of the CoO/GHSs and the GHSs-etched.

**Figure 4:**
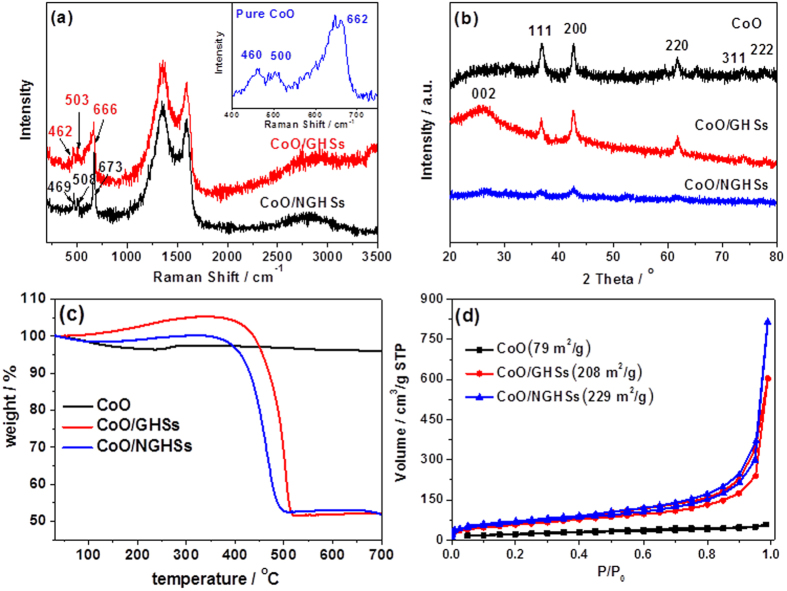
(**a**) Raman spectrum of the CoO/NGHSs and the CoO/GHSs. The inset is the Raman spectrum of the pure CoO solid. (**b**) XRD spectrum and (**c**) TGA curves of the pure CoO solid, the CoO/GHSs and the CoO/NGHSs. (**d**) Nitrogen adsorption/desorption isotherms of the pure CoO solid, the CoO/GHSs, and the CoO/NGHSs. Their corresponding specific surface areas are given in the figure.

The XPS survey spectra in Fig. 2 show that the CoO/NGHSs consist of Co, O, N and C. The presence of CoO could be demonstrated by the two peaks locating at binding energies of 780.0 and 795.8 eV (Fig. 3a), corresponding to Co 2p3/2 and Co 2p1/2, respectively. The prominent shakeup satellite peaks at 786.8 and 802.6 eV in the Co 2p spectrum suggests the domination of the CoO phase (Deconvoluted Co 2p of the CoO/NGHSs is shown in Fig. S3). This is well consistent with the Raman spectra shown in Fig. 4a, in which the formation of CoO could be clearly demonstrated by the peaks at 469, 508, and 673 cm^−1^, assignable to active vibrational modes E_g_, F_2g_, and A_1g_ of spinel oxide CoO, respectively. To confirm the presence of the NGHSs, the spectra deconvolution of C 1 s and N 1 s is done. Figure 3b shows the presence of six different peaks in the deconvoluted spectrum of C 1 s, corresponding to the graphitic C, C-N, C-O, C=O/C=N, O=C-O and the π–π^*^ shakeup satellite peak, respectively. The dominance of the graphitic C, as demonstrated in Fig. 3b, suggests the presence of the graphitic structure. This could be further demonstrated by the Raman spectrum of the CoO/NGHSs shown in Fig. 4a, where two prominent peaks ascribable to the G (associated with the tangential stretching mode of highly ordered pyrolytic graphite) and D (arising from the disordered hybridization carbon) bands could be clearly visualized. In analogy with the C 1s, the deconvoluted N 1s peak in Fig. 3c shows the existence of four nitrogen-containing components, corresponding to pyridinic (397.9 eV), pyrrolic (399.2 eV), quaternary (400.3 eV), and oxidized (403.3 eV) type N-functionalities, respectively. These results are in good agreement with NG reported previously^39^,^40^,^41^, indicating the incorporation of NG in the CoO/NGHSs. In the CoO/NGHSs, NG is indeed aligned in a microspherical structure as demonstrated by the TEM and SEM images shown in Fig. 1. Table 1 summarizes of the relative atomic percentages of nitrogen- and carbon-containing groups in the CoO/NGHSs and their associated peak binding energies based on the XPS analyses.

The XRD pattern of the CoO/NGHSs in Fig. 4b further demonstrates the presence of the CoO nanoparticles, which exhibit a rock salt structure. The size of the CoO nanoparticles calculated from the Debye-Scherrer equation is ∼7.35 nm, close to the average size of the CoO nanoparticles measured from the TEM image shown in Fig. 1a. Although the CoO nanoparticles grown in the absence of the supports or in the presence of the GHSs also exhibit a rock salt structure (as shown in Fig. 4b), their average sizes calculated from the Debye-Scherrer equation are much larger than 15 nm. This indicates that the use of the NGHSs would facilitate the growth of the CoO nanoparticles with smaller sizes, which is consistent with the results of the SEM and TEM images shown in Fig. 1d,e.

To further clarify the structural characteristics of the CoO/NGHSs, their TGA analysis was performed. For comparison, the TGA analyses of the pure CoO solid grown in the absence of the substrate and the CoO/GHSs were also carried out. Figure 4c shows that the TGA curve of the pure CoO solid exhibits a weight loss at the temperature below 220 °C and a slight weight increase at the temperature range from 220 to 330 °C, which could be attributed to the evaporation of physically adsorbed or intercalated water in the CoO aggregate (the significant aggregation of the CoO nanoparticles may lead to the incorporation of water inside the CoO solid) and the oxidation of the CoO nanoparticles, respectively. The TGA curve of the CoO/GHSs exhibits no weight loss corresponding to the evaporation of water, since the hydrophobic nature of the GHSs does not favor the surface adsorption of water molecules. In addition, the relatively improved CoO dispersity excludes the intercalation of water in the CoO aggregates. Indeed, as shown in Fig. 4c, the TGA curve of the CoO/GHSs exhibits a weight increase at the temperature range from 130 to 340 °C and a significant weight loss at the temperature range of 340–530 °C, which could be attributed to the oxidation of the CoO nanoparticles and the subsequent decomposition of the GHSs. The TGA curve of the CoO/NGHSs also exhibits a profile similar to that of the CoO/GHSs at the relatively higher temperature, with a weight increase at the temperature range from 130 to 340 °C and a significant weight loss at the temperature range of 320–510 °C, corresponding to the oxidation of the CoO nanoparticles and the decomposition of the NGHSs. The appearance of the weight loss at the temperature below 130 °C could be attributed to the loss of the physically adsorbed water. This indicates the higher adsorption capability of the CoO/NGHSs towards water in comparison to the CoO/GHSs due to their nitrogen doped structure. The occurrence of the weight loss corresponding to the decomposition of the NGHSs in the CoO/NGHSs at the relatively lower temperature, in comparison to the decomposition of the GHSs in the CoO/GHSs, suggests the doping of nitrogen would decrease the thermal stability of the graphitic structure, consistent with the fact that the doping of nitrogen would decrease the conjugation of the graphitic structure of the NGHSs and produce more defects and edge sites into their sheet plates. Based on the TGA results shown in Fig. 4c, it can be extracted that the relative weight percentage of CoO in the CoO/NGHSs is 47.8%, which is comparable to that in the CoO/GHSs (47.0%) (The detailed calculations of the relative weight percentages of CoO in the CoO/GHSs and the CoO/NGHSs are given in the Supporting Information, Fig. S4). This reveals that the use of the NGHSs does not change the chemical yield of the CoO nanoparticles, but decreases its particles sizes, since these NGHSs could provide the strong anchoring points for the CoO nanoparticle growth.

Figure 4d shows the nitrogen adsorption-desorption isotherms of the CoO/NGHSs measured at 77 K, which display a type IV adsorption–desorption behavior with a H3-type hysteresis loop. The rapid uptake of N_2_ in the p/p_0_ region of 0 to 0.04 suggests the presence of micropores and mesopores, which could be formed from the layered stacking of NG in the wall of the NGHSs and the random stacking of the CoO/NGHSs. The relative larger amount of N_2_ uptake at higher pressure (p/p_0_ > 0.9) as indicated in Fig. 4d gives a clear evidence supporting the presence of a larger fraction of macropores, which is well consistent with the specific structure of the CoO/NGHSs that consists of the hollow structure of the NGHSs. The specific surface area of the CoO/NGHSs estimated using the multi-point Brunauer–Emmett–Teller (BET) method is 229 m^2^/g, larger than those of the pure CoO solid (79 m^2^/g) and the CoO/GHSs (208 m^2^/g). This indicates that the presence of the NGHSs, which facilitates the formation of the CoO nanoparticles with smaller sizes and better dispersity, could increase the specific surface area of the CoO/NGHSs.

**Figure 5:**
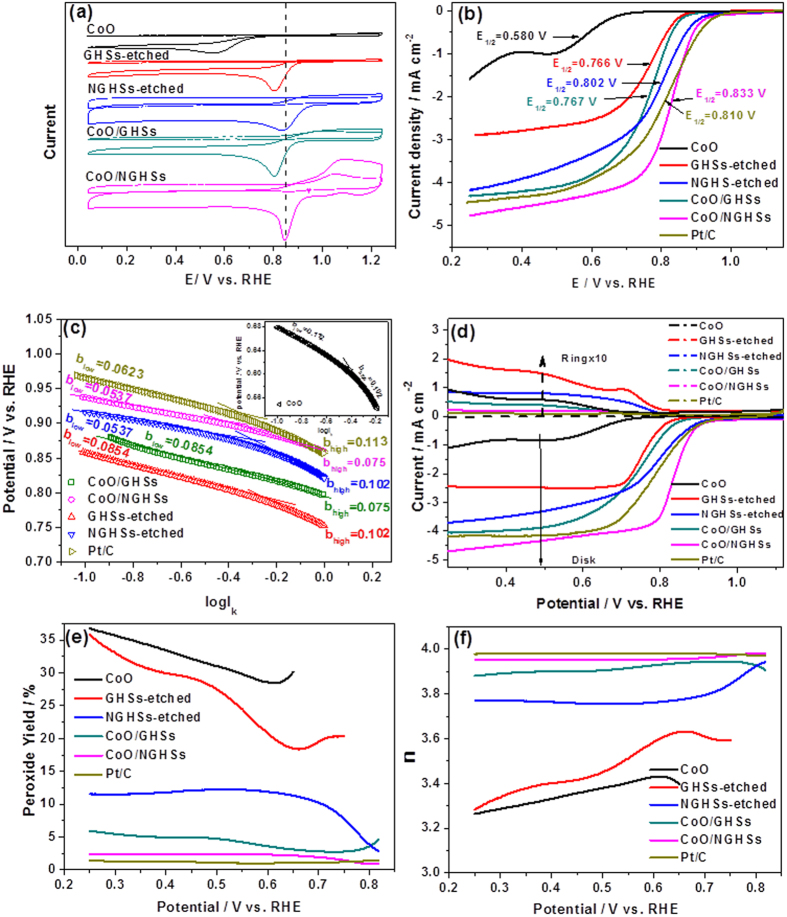
(**a**) CV curves of the pure CoO solid, the CoO/GHSs, the CoO/NGHSs, the GHSs-etched and the NGHSs-etched in O_2_ (solid) or N_2_ (dash) saturated 0.1 M KOH solution at a scan rate of 5 mV/s. (**b**) LSV curves for the ORR on the pure CoO solid, the CoO/GHSs, the CoO/NGHSs, the GHSs-etched, the NGHSs-etched, the Pt/C 20 wt.% in an O_2_-saturated 0.1 M KOH solution at a scan rate of 5 mV/s. (**c**) Tafel plots of the pure CoO solid, the CoO/GHSs, the CoO/NGHSs, the GHSs-etched, the NGHSs-etched, and the Pt/C 20 wt.%, derived by the mass-transport correction of their corresponding RDE data (see the Supporting Information). (**d**) RRDEs voltammograms of the pure CoO solid, the CoO/GHSs, the CoO/NGHSs, the GHSs-etched, the NGHSs-etched, the Pt/C 20 wt.% in O_2_-saturated 0.1 M KOH at 1600 rpm. The disk potential was scanned at the rate of 5 mV/s, and the ring potential was constant at 1.3 V vs. RHE. (**e**) Peroxide yield and (**f**) electron transfer number (n) of the pure CoO solid, the CoO/GHSs, the CoO/NGHSs, the GHSs-etched, the NGHSs-etched, the Pt/C 20 wt.% at various potentials obtained based on their corresponding RRDE data in (**d**). Catalyst loading was ∼0.20 mg/cm^2^ for all the samples.

To evaluate the catalytic behaviors of the CoO/NGHSs for the ORR, their cyclic voltammograms (CVs) in an aqueous solution of 0.1 M KOH saturated with O_2_ or N_2_ were first measured. Figure 5a shows that the CV of the CoO/NGHSs in the O_2_ saturated solution exhibits a well-defined cathodic peak, corresponding to the reduction of oxygen, while no cathodic peak at this position could be observed in their CV in the N_2_ saturated solution. This clearly demonstrates the electrocatalytic activity of the CoO/NGHSs for the ORR. The catalytic activity of the CoO/NGHSs for the ORR could further be demonstrated by their linear sweep voltammogram (LSV) in Fig. 5b, where the voltammetric current corresponding to the reduction of the oxygen could clearly be identified. Indeed, as shown in Fig. 5b, the CoO/NGHSs could even exhibit more positive half-wave potential (E_1/2_ = 0.833 V, the potential at which the current is half of the limiting current density) and higher limiting current density for the ORR than the commercial JM Pt/C 20 wt.% (E_1/2_ = 0.810 V, which is comparable to those reported in the literature^42^,^43^,^44^,^45^,^46^), although their onset potentials for the ORR appear at the relatively comparable position. This indicates that the CoO/NGHSs are the more active catalysts for the ORR than the JM Pt/C 20 wt.%. Up to now, although a significant amount of catalysts has been demonstrated to be active for the ORR^47^,^48^,^49^, those with catalytic activities higher than the JM Pt/C 20 wt.% have less been reported. The present work is therefore of great interest since it provides a simple method to synthesize the catalysts with significantly higher ORR activity.

On the basis of the structural information obtained above, we would attribute the small size of the CoO nanoparticles and the higher specific surface area of the CoO/NGHSs to two of the possible reasons leading to the higher catalytic activity of the CoO/NGHSs. This could be demonstrated by the results in Fig. 5a,b, which shows that although both the pure CoO solid and the CoO/GHSs are catalytically active for the ORR, their catalytic activities are indeed much lower than that of the CoO/NGHSs due to their larger CoO nanoparticle sizes and relatively lower specific surface areas. The small size of the CoO nanoparticles and the higher specific surface area of the CoO/NGHSs would facilitate more active materials accessible to the ORR and increase the solid/electrolyte interface area during the electrochemical reaction, both of which could enhance the electrocatalytic activity of the CoO/NGHSs. This could further be demonstrated by the higher electroactive surface area of the CoO/NGHSs in comparison to those of the pure CoO solid and the CoO/GHSs, as determined by their CVs in the Fe(CN)_6_^3−^/Fe(CN)_6_^4−^ solution shown in Fig. S5.

The electrocatalytic reaction involves electron transfer, which requires catalysts to be electrically conductive to achieve high activity. The electric conductivity measurements show that the CoO/NGHSs have conductivity of ∼76.5 S/m, which is higher than that of the CoO solid (∼3.4 S/m), the CoO/GHSs (∼50.7 S/m) and the Pt/C 20 wt.% (∼69.6 S/m). This indicates that the high electrical conductivity could be an additional reason leading to the enhanced catalytic activity of the CoO/NGHSs, since it facilitates the transfer of electron generated during the ORR.

Previous work has reported that the distinct catalytic properties of the composite materials combining two or more components may originate from the collective interactions between the components^50^. To demonstrate that there exists the interaction between CoO and NGHSs in the CoO/NGHSs, the XPS spectra of the NGHSs obtained by the etching of the CoO/NGHSs with a HCl solution (NGHSs-etched) were measured. As shown in Fig. 2b, the XPS survey spectrum of the obtained NGHSs-etched shows the presence of only elemental C, O and N with the relatively peak intensity of oxygen greatly reduced, indicating that CoO has been well removed from the CoO/NGHSs through the etching. The high-resolution spectra in Fig. 3b,c show that the C 1s and N 1s peaks of the NGHSs-etched possess the comparable shapes to those of the CoO/NGHSs, but with the positions appearing at the relatively higher binding energy, which strongly suggests the presence of the strong interaction between CoO and NGHSs in the CoO/NGHSs with a possible electron transfer from CoO to NGHSs. This is different from the CoO/GHSs, in which the interaction between CoO and GHSs is not detectable. As shown in Fig. 3d, the high-resolution C1s spectrum of the GHSs obtained from the etching of the CoO/GHSs with the HCl solution (GHSs-etched) exhibits a peak resembling to that of the CoO/GHSs and centered at the position similar to that of CoO/GHSs, indicating the insignificant interaction between CoO and GHSs in the CoO/GHSs. Indeed, the strong interaction between CoO and NGHSs in the CoO/NGHSs might also be demonstrated from the appearance of the Co 2p XPS peaks of CoO in the CoO/NGHSs at the relatively higher binding energies and the emergence of the E_g_, F_2g_, and A_1g_ Raman modes of CoO in the CoO/NGHSs at the higher wavenumbers in comparison to those in the CoO/GHSs, as shown in Figs 3a and 4a, respectively. The results presented here suggest that the nitrogen doped structure could not only facilitate the formation of the CoO nanoparticles with good monodispersity on the surface of the NGHSs, but also promote the interaction between CoO and NGHSs.

Figure 3b,c show the deconvoluted XPS spectra of C 1s and N 1s of the NGHSs-etched, which demonstrate the existence of the nitrogen- and oxygen-containing components with the numbers and types similar to those in the CoO/NGHSs. Table 1 indicates that the relative percentages of the nitrogen or carbon-containing components have been changed after the CoO nanoparticle removal. This might be attributed to the facts that the removal of the CoO nanoparticles would make functional groups, which are initially covered by the CoO nanoparticles, detectable by the XPS spectroscopy. The great increase in the relative percentage of the quaternary nitrogen-containing components suggests that most of the CoO nanoparticles are anchored on the quaternary nitrogen-containing components. It is well consistent with the results shown above that the presence of the NGHSs would promote the formation of the CoO nanoparticles with smaller sizes and better dispersity, since the quaternary nitrogen with a relatively higher electron density^51^,^52^, which is usually located in the interior of the graphitic plane of the NG^51^,^52^, could be used as the anchoring points for the nucleation and growth of the CoO nanoparticles through its coordination and electrostatic interaction with the Co^2+^ ions and the NG plane could well support the deposition of the CoO nanoparticles. The decrease in the relative percentages of pyridinic, pyrrolic, and oxidized type nitrogen-containing components may suggest that these nitrogen-containing components are not well favorable for the CoO nucleation and growth, since the edge and defect site located pyridinic and pyrrolic type nitrogen lacks the mechanically stable supporting planes for the growth of the CoO nanoparticles due to the high gravity of the CoO nanoparticles and the highly pliable nature of the graphitic structure (the pyridinic and pyrrolic type nitrogen components are usually doped in the edge and defect sites of graphene^51^,^52^,^53^,^54^), while oxidized nitrogen with a low electron density is not favored for its coordination with the Co^2+^ ions. The slight increases in the relative percentages of the oxygen bonded carbon-containing components, as shown in Table 1, may suggest the anchoring of a small number of the CoO nanoparticles on the oxygen-containing groups. This is similar to the CoO/GHSs, where the slight increases in the relative percentages of the oxygen bonded carbon-containing components have been observed after the CoO removal. However, due to the relatively low interaction between the oxygen containing groups and CoO, the significant aggregation of the CoO nanoparticles occurs when the GHSs in the absence of the nitrogen doped structure are used for their growth, as demonstrated by the TEM image of the CoO/GHSs shown in Fig. 1d.

Figure 5a shows that the NGHSs-etched are also catalytically active for the ORR, similar to the NG and the NGHSs reported previously^55^,^56^,^57^. However, their catalytic activity is much lower than that of the CoO/NGHSs, as demonstrated by their less positive half-wave potential and lower limiting current density shown in Fig. 5b. Along with the low catalytic activity of the pure CoO solid, it makes us believe that the interaction between CoO and NGHSs plays an important role in the higher catalytic activity of the CoO/NGHSs. This could further be demonstrated by the comparable half-wave potential of the ORR for the GHSs-etched and the CoO/GHSs, indicating that the insignificant interaction between CoO and GHSs would not change the relative reactivity of the GHSs-etched and the CoO/GHSs for the ORR. The improved current density of the ORR for the CoO/GHSs in comparison to that for the GHSs-etched (shown in Fig. 5b) might be attributed to the simple summation of the electrocatalytic activity of the CoO nanoparticles and the GHSs or the improved dispersity of the CoO nanoparticles, which facilitates more CoO accessible to the ORR.

To obtain information about the oxygen adsorption mechanism on the electrocatalysts, diffusion-corrected Tafel curves were plotted by eliminating the impact of the mass transport on the adsorption kinetics of oxygen (see Supporting Information for details). Figure 5c shows that the Tafel plot of the Pt/C 20 wt.% exhibit two distinct regions with the Tafel slopes of 62.3 mV dec^−1^ and 113 mV dec^−1^ at the low and high current densities, corresponding to the Temkin and Langmuir adsorption of oxygen, respectively. This is in good agreement with those reported previously^58^,^59^. Although the Tafel plot of the CoO/NGHSs also exhibits two distinct regions in the high and low current densities, their slopes are much lower than those of the Pt/C 20 wt.% (the respective Tafel slopes for the CoO/NGHSs are 53.7 and 75 mV dec^−1^ at the low and high current densities), suggesting that the oxygen adsorption on the CoO/NGHSs is more energetically favorable. The lower Tafel slopes indicate that the overpotential increases slowly with current density, which could be an additional reason resulting in the superior ORR activity of the CoO/NGHSs.

To understand the underlying physics leading to the lower Tafel slopes for the CoO/NGHSs, the diffusion-corrected Tafel curves for the CoO/GHSs, the NGHSs-etched and the GHSs-etched were also plotted. Figure 5c shows that the CoO/NGHSs and the NGHSs-etched have similar Tafel slopes at the low current density region, which may suggest that the Tafel slope for the CoO/NGHSs at the low current density region is determined by the NGHSs. This is similar to the case of the CoO/GHSs and the GHSs-etched, in which the similarity of their Tafel slopes at the low current density region could also be observed, as shown in Fig. 5c. The slightly lower Tafel slope for the CoO/NGHSs at the low current density region in comparison to that for the CoO/GHSs could be attributed to their presence of nitrogen doped graphitic structure. Previous reports have demonstrated that the interaction between ^3^O_2_ and carbon materials was a weak physisorption, while the nitrogen doping would make the charge density of carbon atoms adjacent to the nitrogen dopants energetically favorable for the adsorption of ^3^O_2_^60^,^61^,^62^. Oxygen dissociation was then facilitated by the charge transfer associated with carbon atoms adjacent to the nitrogen dopants due to the reduction in the dissociation and activation barriers^53^,^63^,^64^. This suggests that the presence of the NGHSs gives a big contribution on the high catalytic activity of the CoO/NGHSs. In addition, the lower Tafel slope of the CoO/NGHSs at the high current density region in comparison to the NGHSs-etched indicates that the presence of the CoO nanoparticles could also lead to the low Tafel slope of the CoO/NGHSs. Based on these, we would speculate that the excellent electrocatalytic activity of the CoO/NGHSs could be attributed to the lower O_2_ adsorption and dissociation energy resulting from their nitrogen-doping and CoO deposited structure, since both of them could lower the Tafel slopes.

The results shown above make us believe that the superior ORR performance of the CoO/NGHSs possibly arises from the following reasons: (1) the nitrogen doped structure of the NGHSs, (2) the small size of the CoO nanoparticles, (3) the higher specific and electroactive surface areas of the CoO/NGHSs due to their porous structure, (4) the good electric conductivity of the CoO/NGHSs, and (5) the strong interaction between the CoO nanoparticles and the NGHSs, and (6) the lower O_2_ adsorption and dissociation energy resulting from the nitrogen doping and the CoO deposited structure. To gain insight into the reduction pathways of oxygen by the catalysts, their voltammograms measured on the rotating ring-dick electrodes (RRDEs) were recorded. Figure 5d shows the obtained RRDE voltammograms, in which the disk current associated with the reduction of oxygen and the ring current associated with the peroxide (H_2_O_2_) oxidation are presented. In good agreement with the LSV results shown in Fig. 5b, the higher catalytic activity of the CoO/NGHSs than those of the pure CoO solid, the CoO/GHSs, the GHSs-etched, the NGHSs-etched, and the Pt/C 20 wt.% could further be demonstrated by their more positive half-wave potential and the higher limiting current for the ORR. Based on the RRDE voltammograms, the number of the electron transferred (n) and the yield of H_2_O_2_ (*H*_*2*_*O*_*2*_*%*) produced during the ORR could be determined (see Supporting Information for details). Figure 5e shows that the electron transfer number for the ORR by the CoO/NGHSs is higher than 3.95 over the measured potential range, indicating that the ORR catalyzed by the CoO/NGHSs mainly proceeds via the four-electron pathway. This could further be demonstrated by their negligible H_2_O_2_ production yield shown in Fig. 5f. The electron transfer numbers for the pure CoO solid, the CoO/GHSs, the GHSs-etched, and the NGHSs-etched are lower than that of the CoO/NGHSs, suggesting that the two-electron pathway plays an increasing role in the ORR for those catalysts. This is well consistent with their higher H_2_O_2_ production yield shown in Fig. 5f and the results obtained from the Koutecky-Levich plots based on RDE measurements (Fig. S6). The higher electron transfer number and lower H_2_O_2_ production yield, as shown in Fig. 5e,f, further demonstrate that the CoO/NGHSs are more efficient catalysts than the pure CoO solid, the CoO/GHSs, the GHSs-etched, and the NGHSs-etched. The most interesting is that although the CoO/NGHSs exhibits slightly higher H_2_O_2_ production yield and lower electron transfer number than those of the Pt/C 20 wt.%, their electrocatalytic activity is higher than that of the Pt/C 20 wt.%. This might be due to their lower O_2_ adsorption and dissociation energy for oxygen reduction demonstrated above and much higher electroactive surface area confirmed in the Supporting Information.

**Figure 6:**
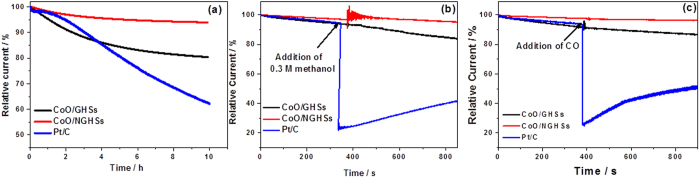
(**a**) Durability evaluation of the CoO/GHSs, the CoO/NGHSs, and the Pt/C 20 wt.% for 10 hs at 0.75 V vs. RHE in an O_2_-saturated 0.1 M KOH solution. (**b**) Chronoamperometric response for the ORR at the electrodes of the CoO/GHSs, the CoO/NGHSs, and the Pt/C 20 wt.% by introducing 0.3 M methanol into the electrolyte at 380 s, and (**c**) Chronoamperometric response for the ORR at the electrodes of the CoO/GHSs, the CoO/NGHSs, and the Pt/C 20 wt.% by introducing additional CO into the electrolyte at 380 s.

To further verify that the CoO/NGHSs can be used as the efficient catalysts for the ORR, their stability and durability toward methanol crossover and carbon monoxide (CO) poisoning were measured. Figure 6a shows that the CoO/NGHSs can remain highly efficient for the ORR with loss of only 6.2% of their original activity over 10 h of the ORR. The introduction of methanol and CO shows no influence on the catalytic activity of the CoO/NGHSs. The stability of the CoO/NGHSs and their durability towards methanol crossover and CO poisoning are much higher than that of the Pt/C 20 wt.%. As shown in Fig. 6a, a loss of over 38% of the original activity of the Pt/C 20 wt.% could be observed after 10 h of the ORR, and the introduction of methanol and CO shows even greater losses of its activity, due to the blockage of active sites on the Pt nanoparticles by the adsorption of CO or the methanol oxidation products^65^,^66^,^67^. Worthnoting is that although the introduction of methanol or CO also has no effects on the oxygen reduction activities of the CoO/GHSs, they are indeed less stable compared to the CoO/NGHSs, as shown in Fig. 6b,c. This indicates that the strong interaction between CoO and NGHSs could also make the CoO/NGHSs more stable toward ORR. The TEM and SEM images in Fig. S7a,b show no noticeable changes in the morphology of the CoO/NGHSs, including the size and dispersity of the CoO nanoparticles, after 10 h of the ORR at 0.75 V vs. RHE in an O_2_-saturated 0.1 M KOH solution. This is contrast to the TEM and SEM images of the CoO/GHSs (Fig. S7c,d), in which a decrease in the number of the CoO aggregates on the surface of GHSs could be clearly observed after 10 h of the ORR. The weak interaction between CoO and GHSs makes the CoO aggregates easily detachable from the GHSs supports.

**Figure 7:**
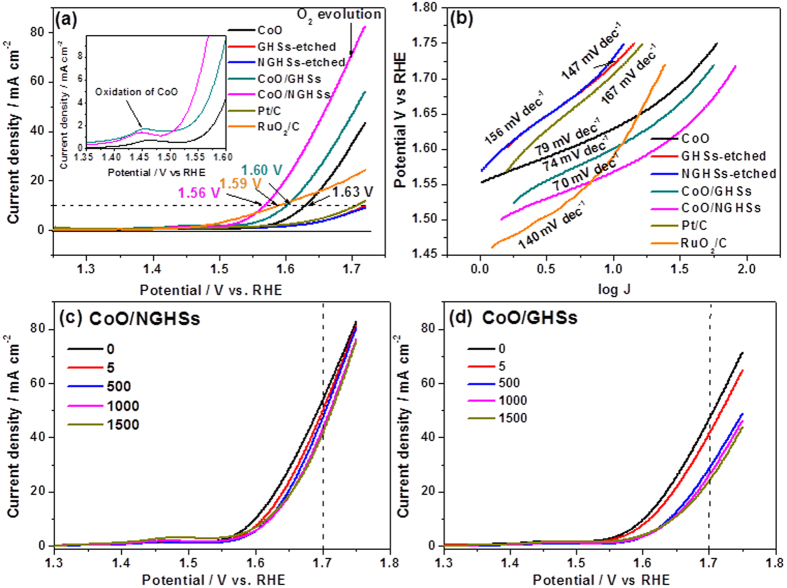
(**a**) Oxygen evolution current densities of the pure CoO solid, the CoO/NGHSs, the CoO/GHSs, the GHSs-etched, the NGHSs-etched, the Pt/C 20 wt.%, and RuO_2_/C loaded onto the RDE electrodes (catalyst loading of ∼0.2 mg cm^−2^) measured in 1.0 M KOH with a sweep rate of 5 mV/s. (**b**) OER Tafel plots of the pure CoO solid, the CoO/NGHSs, the CoO/GHSs, the GHSs-etched, the NGHSs-etched, the Pt/C 20 wt.%, and RuO_2_/C and their corresponding Tafel slopes are given in the figure. (**c**,**d**) LSVs of the CoO/NGHSs and the CoO/GHSs in the N_2_-saturated 1.0 M KOH electrolyte (catalyst loading 0.20 mg/cm^2^ for all samples). Cycles were swept between 1.25 V and 1.75 V at 0.2 V/s. The numbers of the catalysts cycled are shown in the figures. All the data shown in Fig. 7 were IR-corrected using the solution resistance measured by electrochemical impedance spectroscopy at the open-circuit potential.

To demonstrate that the CoO/NGHSs are also catalytically active for the OER, the LSV of the catalysts loaded on the glassy carbon electrode in the N_2_–saturated 1.0 M KOH electrolyte was measured in the water oxidation potential regime, as shown in Fig. 7a. The appearance of the high current density at the potential >1.4 V vs. RHE indicates the electrocatalytic activity of the CoO/NGHSs for the OER. Although the pure CoO solid, the CoO/GHSs, the GHSs-etched, the NGHSs-etched, and the Pt/C 20 wt.% are also electrocatalytically active for the OER, their catalytic activities are much lower than that of the CoO/NGHSs, as indicated by their relatively more positive onset potentials for the OER and larger overpotentials at a current density of 10 mA/cm^2^ (Fig. 7a). Worthnoting is that although the OER onset potential of the CoO/NGHSs is more positive than that of the conventional RuO_2_/C, their overpotential that reaches to a current density of 10 mA cm^−2^ is smaller than that of the RuO_2_/C. This further demonstrates that the CoO/NGHSs are the efficient catalysts for the OER. The lower OER catalytic activities for the pure CoO solid, the GHSs-etched, the NGHSs-etched, the CoO/GHSs and the Pt/C 20 wt.% could also be demonstrated by their high OER Tafel slopes in comparison to that of the CoO/NGHSs, as shown in Fig. 7b. These results might suggest that the factors, such as the small size of the CoO nanoparticles, the higher specific and electroactive surface areas of the CoO/NGHSs, the good electric conductivity of the CoO/NGHSs, the strong interaction between the CoO nanoparticles and the NGHSs, etc., which lead the CoO/NGHSs to the high activity for the ORR could also make them highly active for the OER. The inset in Fig. 7a shows a CoO oxidation peak at 1.44 V, which is commonly observable in the Co-based catalysts^68^,^69^. The emergence of their CoO oxidation peak at the low potential in comparison to those of the pure CoO solid and the CoO/GHSs could be attributed to the relatively smaller size of the CoO nanoparticles, making them more oxidizable. This is in good agreement with the results of the TEM image shown above. The most interesting is that the OER overpotential of the CoO/NGHSs at the current density of 10 mA/cm^2^ is ~0.33 V (with respect to the theoretical OER potential of 1.23 V), which is comparable to the performance of the best OER catalysts reported previously, such as Ni(OH)_2_^70^, Co_3_O_4_^23^, Co_3_O_4_-rmGO^28^, etc. This indicates that the CoO/NGHSs are among the most efficient OER catalysts reported to date.

The durability tests further demonstrate that the CoO/NGHSs could be used as the efficient catalysts for the OER. The LSVs of the catalysts in the N_2_–saturated 1.0 M KOH electrolyte shown in Fig. 7c demonstrate that the CoO/NGHSs could remain highly active for the OER with a only loss of ~23% of its original current density at 1.7 V after 1500 cycles, although a large current decrease of 9.3% could be observed in the initial 5 cycles presumably due to the blockage of the active sites by the gradually accumulated evolved O_2_ bubbles. Indeed, as shown in Fig. 7a, the OER current for the CoO/NGHSs does not decrease significantly after 1000 cycles. These results clearly suggest the good stability of the CoO/NGHSs for the OER, similar to those of the electrocatalysts reported previously^28^,^68^. The stability of the CoO/NGHSs is much higher than that of the CoO/GHSs. As shown in Fig. 7d, besides a higher current decrease of 12.3% in the initial 5 cycles, the CoO/GHSs also undergo a much higher decrease in the current density (~49%) at 1.7 V after 1500 cycles. The higher stability of the CoO/NGHSs for the OER may suggest that the strong interaction between CoO and NGHSs could also make them highly stable during the catalytic reaction, which could be demonstrated by their TEM and SEM images in Fig. S7e,f, where no noticeable changes in the morphology of the CoO/NGHSs could be observed after the OER. This is different from the CoO/GHSs, in which the weak interaction between CoO and GHSs has led to a significant loss of their catalytic activity during the OER, due to the detachment of the CoO aggregates from the GHSs during the OER, as demonstrated by their SEM and TEM images in Fig. S7g,h.

## Conclusions

In summary, the CoO/NGHSs have been developed by the growth of the CoO nanoparticles on the NGHSs. The presence of the nitrogen doped structure plays an important role in the formation of the CoO nanoparticles with good monodispersity. Most of the CoO nanoparticles might be anchored on the quaternary nitrogen since the coordination and electrostatic interactions of the high electron density of the quaternary nitrogen with the Co^2+^ ions facilitates the nucleation and growth of the CoO nanoparticles. The XPS results show the existence of the strong interaction between CoO and NGHSs with a possible electron transfer from CoO to NGHSs. The CoO/NGHSs shows superb catalytic activities and excellent stability for both the ORR and the OER with the ORR activity and stability higher than the commercial Pt/C 20 wt.% and the OER activity and stability comparable to the most efficient OER catalysts reported to date. This clearly suggests the potential use of the CoO/NGHSs as efficient bifunctional catalysts for the ORR and the OER. In addition, the results presented here make clear that the presence of the quaternary nitrogen could not only facilitate the formation of the CoO nanoparticles with good monodispersity, but also promote the strong interaction between CoO and NGHSs, which are crucial to obtain the CoO/NGHSs with high electrocatalytic activity and good stability for the ORR and the OER. These findings are also beneficial to develop other NG supported TMO nanoparticles as the catalysts for various applications.

## Methods

### Materials Synthesis

The synthesis of the CoO/NGHSs was carried by three steps: In the first step, CoSO_4_·7H_2_O and urea was dispersed in alcohol–water solution and NGHSs was dissolved in water by sonication. Then the two solutions were mixed by agitation, followed by adding ammonia solution drop by drop. The reaction was stirred for 30 min. In the second step, the reaction mixture from the first step was transferred to an autoclave for hydrothermal reaction at 120 °C for 12 h. In the third step, the obtained composite was dried at 60 °C overnight, followed by calcination at 450 °C in N_2_ for 3 h. The synthesis of the CoO/GHSs was carried out using the similar procedure used for the synthesis of the CoO/NGHSs with the NGHSs substituted by an equimolecular amount of GHSs. The pure CoO solid was prepared by the same process without using the NGHSs and the GHSs. See Supporting Information for detailed experimental procedures.

### Electrochemical measurement

Cyclic voltammetry (CV), RDE and RRDE were conducted with a CHI 750E electrochemical workstation (CH Instruments, Chenhua Co., China) in a conventional three-electrode cell, with a platinum gauze as the counter electrode, a saturated calomel electrode (SCE) as the reference electrode, and a glassy carbon electrode loaded with various catalysts as the working electrode. 40 μg of sample was loaded on the glassy carbon working electrode, rotating disk electrode and rotating ring-disk electrode, respectively. In order to ensure the repeatability of the test results, all the tests were performed 3–5 times. See Supporting Information for detailed experimental procedures.

### Characterization

Scanning electron microscopic (SEM) images of the obtained samples were acquired on a field-emission scanning electron microscope (S-4800, Hitachi) at an operation voltage of 20.0 kV. TEM measurements were conducted on a JEM-2100F high-resolution transmission electron microscope with an accelerating voltage of 200 kV. Powder X-ray diffraction (XRD) patterns were recorded with a Bruker D8-Advance diffractometer using Cu Kα radiation. The chemical compositions of the samples were determined by X-ray photoelectron spectroscopy (XPS) on a VG ESCALAB 250 spectrometer (Thermo Electron, U.K.), using an Al Kα X-ray source (1486 eV). Thermogravimetric analysis (TGA) was performed on a METTLER instruments under an O_2_ atmosphere at a heating rate of 5 °C/min. Brunauer−Emmet−Teller (BET) surface area was determined by using an AUTOSORB-IQ-MP instrument with nitrogen adsorption at 77 K using the Barrett−Joyner−Halenda (BJH) method. Raman spectra were recorded on a RENISHAW inVia instrument with an Ar laser source of 488 nm in a macroscopic configuration.

## Additional Information

**How to cite this article**: Jiang, Z.-J. and Jiang, Z. Interaction Induced High Catalytic Activities of CoO Nanoparticles Grown on Nitrogen-Doped Hollow Graphene Microspheres for Oxygen Reduction and Evolution Reactions. *Sci. Rep.* **6**, 27081; doi: 10.1038/srep27081 (2016).

## Supplementary Material

Supplementary Information

## Figures and Tables

**Table 1:** Peak assignments for the carbon- and nitrogen-containing components for the CoO/NGHSs, the CoO/GHSs, the NGHSs-etched and the GHSs-etched and their relative percentages.

Peak	Binding energy (eV)/fraction of species (%)				
	CoO/NGHSs	NGHSs-etched	CoO/GHSs	GHSs-etched	Assignment
C1s	284.19/63.1	284.58/55.9	284.60/73.3	284.65/69.2	sp2 C–C or C–H
	285.45/18.0	285.75/20.0	–	–	C–N
	286.50/7.9	286.75/10.1	286.05/15.6	286.10/16.1	C–O
	287.60/3.9	287.80/4.6	287.90/4.7	287.90/7.1	C=O/C=N
	289.35/5.4	289.50/6.3	289.90/5.1	289.90/4.9	O=C–O
	291.50/1.6	291.70/3.1	291.90/1.3	292.00/2.8	π−π*
N1s	397.82/37.5	398.04/33.9	–	–	pyridinic
	399.00/21.2	399.32/19.2	–	–	pyrrolic
	400.25/27.2	400.41/36.1	–	–	quaternary
	402.70/14.1	403.30/10.8	–	–	N-oxide
