# Supplementary material for: Interaction Induced High Catalytic Activities of CoO Nanoparticles Grown on Nitrogen-Doped Hollow Graphene Microspheres for Oxygen Reduction and Evolution Reactions
Source: Sci Rep. 2016 Jun 3;6:27081. doi: 10.1038/srep27081 (PMC4891770; doi:10.1038/srep27081)
Supplement: Supplementary Information [file srep27081-s1.pdf]

Supporting information

Interaction Induced High Catalytic Activities of CoO Nanoparticles Grown on Nitrogen-Doped Hollow Graphene Microspheres for Oxygen Reduction and Evolution Reactions

Zhong-Jie Jiang[1,*,+] & Zhongqing Jiang[2,+]

[1] New Energy Research Institute, College of Environment and Energy, South China University of Technology, Guangzhou 510006, Guangdong, China.

[2] Department of Chemical Engineering, Ningbo University of Technology, Ningbo 315016, Zhejiang, China.

[+]these authors contributed equally to this work.

[*] **Correspondence.** Professor Z-J Jiang, New Energy Research Institute, College of Environment and Energy, South China University of Technology, Guangzhou 510006, Guangdong, China.

E-mail: zhongjiejiang1978@hotmail.com or eszjiang@scut.edu.cn

Tel: +86-020-39381202.

1. Experimental section

1.1 Materials and reagents

Flake graphite (325 meshes) was purchased from Alfa Ltd. Concentrated sulphuric acid (H_2SO_4, 95.0～98.0 %), nitric acid (HNO_3, 65.0～68.0 %), methanol (CH_3OH, ≥99.7 %), sodium nitrate ($NaNO_3$, ≥99.0 %), potassium permanganate ($KMnO_4$, ≥99.5 %), hydrochloric acid (HCl, 36.0～38.0 %), hydrogen peroxide aqueous solution (H_2O_2, 30.0%), poly(N-vinyl-2-pyrrolidone), and ethanol (CH_3CH_2OH, ≥99.5%) were obtained from Shanghai Chemical Reagent Co. Ltd. Melamine ($C_3H_6N_6$, 99%), and 2,2'-Azobis(2-methylpropionamide)dihydrochloride (AIBA, 97%) were obtained from aladdin Co. Ltd. The commercially available Johnson Matthey (JM) Pt/C 20 wt. % (Pt loading: 20 wt. % Pt on the carbon black) was purchased from the Johnson Matthey Corp. All the chemicals are used as received without further purification. Deionized (DI) water (H_2O) through Millipore system (Milli-Q®) was used in all the experiments.

1.2 Material synthesis

1.2.1 Preparation of the PS spheres

The positively charged PS spheres used as the sacrificing template for the synthesis of the CoO/NGHSs was fabricated following the procedure reported by Wu et al.[1] Typically, 10.0 g of styrene and 1.5 g of poly(N-vinyl-2-pyrrolidone) were first mixed with 100.0 mL of deionized water in a 500 mL three-neck flask. After stirring for 30 min, 0.26 g of 2,2'-Azobis(2-methylpropionamide)dihydrochloride (AIBA) dissolved in 20 mL of deionized water was injected. The obtained mixture was deoxygenated by nitrogen flow for 30 min and was then heated to 70 ^{o}C. The reaction was lasted for 24 hours. The obtained product was centrifuged with methanol and deionized water and dried in an oven overnight. The ζ-potential measurement showed that the PS spheres had a ζ-potential of 51.6 ± 0.25 mV at pH 5.0-7.0, comparable to the reported value[2].

1.2.2 Preparation of GO and GO solution

GO was synthesized from the flake graphite following the procedure reported by Hummers et al. [3] with a slight modification, as described in our previously published work.[4-6] In a typical synthesis, 2.0 g of graphite and 2.5 g of $NaNO_3$ were first mixed with 150 mL of H_2SO_4 (95%) in a 500 mL flask. The obtained mixture was then stirred for 30.0 min with an ice bath. Under vigorous stirring, 15.0 g of $KMnO_4$ was added under well control to maintain the reaction temperature below 20 °C. The reaction mixture was then stirred overnight at room temperature, followed by addition of 180 mL of H_2O also under vigorous stirring. After that, the reaction temperature was rapidly increased to 98 °C and the mixture were kept at 98 °C for 24 h, leading to a color change from black to yellow. Followed by the addition of 80.0 mL of 30.0% H_2O_2 aqueous solution, the reaction mixture was cooled down to room temperature. The obtained graphene oxide was washed by rinsing and centrifugation with 5.0% HCl and H_2O for several times, and then filtrated and dried under vacuum.

1.2.3 Preparation of the GHSs and NGHSs

The preparation of the NGHSs was carried out as follows: 0.564 g of the positively charged PS spheres synthesized above was added into 80 mL of deionized water containing 128.7 mg negatively charged GO. The obtained mixture was stirred at room temperature for 12 h, which led to the wrapping of GO onto positively charged PS spheres through an electrostatic interaction. After that, 4.0 g of melamine was added into the above GO wrapped PS spheres (GO/PS) solution, and the mixture was stirred again at room temperature for 12 h before it was moved to an oven at 60 °C for 24 h. Through hydrogen bonding between the amine groups in the melamine and the oxygenous groups in GO, ionic bonding between protonated amines and carboxyls, π-π interaction between unoxidized area in GO and triazine rings, melamine could adsorb onto the surface of GO/PS particles. For the doping of N into the graphitic structure of graphene, the melamine adsorbed GO/PS particles were placed in an alumina crucible in a horizontal furnace under a pure nitrogen gas atmosphere, heated to 800 °C with a heating rate of 5 °C min^{-1}, and then calcinated for 1 h.

The synthesis of GHSs was accomplished using the similar procedure used for the synthesis of NGHSs while in the absence of melamine.

1.2.4 Synthesis of the CoO/NGHSs, the CoO/GHSs and the pure CoO solid

The synthesis of the CoO/NGHSs was carried out as follows: First, 0.14 g $CoSO_4 \cdot 7H_2O$ and 0.3 g urea was dispersed in 37.5 mL alcohol–water (1:5, v/v) solution by sonication, and 0.1 g NGHSs was dissolved in 10 mL water. Then the two solutions were mixed by agitation, followed by adding 4 mL 25% ammonia solution drop by drop. After 30 min of stirring, a transparent solution was obtained and then transferred into Teflon-lined stainless steel autoclaves. Next, the autoclave was sealed and heated at 120 °C for 12 h and then allowed to cool to room temperature spontaneously. After the reaction, the composite was collected from the solution, ultrasonicated in deionized water for 1 min and then centrifuged to remove free nanoparticles and residual reactant. The obtained composite was dried at 60 °C overnight, followed by calcination at 450 °C in N_2 for 3 h. The CoO contents were tested by thermogravimetric analysis (TGA).

The synthesis of the CoO/GHSs was carried out using the similar procedure used for the synthesis of the CoO/NGHSs with the NGHSs substituted by an equimolecular amount of GHSs. The pure CoO solid was prepared by the same process without using the NGHSs and the GHSs.

1.3 Electrochemical measurement

1.3.1 Cyclic voltammetry (CV) measurement

To prepare the working electrode, 4.0 mg as-synthesized catalyst was mixed with 87 μL Nafion solution (5.0% Nafion in ethanol), 652 μL DI water, and 261 μL isopropyl alcohol. The mixture was sonicated at least 60 min to form a homogeneous dispersion, and 10.0 μL suspension was loaded onto a glassy carbon electrode of 5 mm in diameter (loading ~ 0.20 mg/cm^2) and then fully dried. For a comparison, the commercially available Johnson Matthey (JM) Pt/C 20 wt. % (Johnson Matthey Corp., Pt loading: 20 wt. % Pt on the carbon) electrode was also prepared. The Pt/C suspension was prepared by dispersing 4.0 mg of the Pt/C powder in 965 μL of ethanol in the presence of 35 μL of 5 wt. % Nafion solution in isopropyl alcohol. The addition of a small amount of Nafion could effectively improve the dispersion of the Pt/C catalyst suspension. The CV measurements were conducted with a CHI 750E electrochemical workstation (CH Instruments, Chenhua Co., China) in a conventional three-electrode cell, with a platinum gauze as the counter electrode, a saturated calomel electrode (SCE) as the reference electrode, and a glassy carbon electrode loaded with various catalysts as the working electrode. Electrolyte was saturated with oxygen by bubbling O_2 prior to the start of each experiment. A flow of O_2 was maintained over the electrolyte during the recording of CVs to ensure its continued O_2 saturation. The working electrode was cycled at least 10 times before data were recorded at a scan rate of 5 mV/s. In control experiments, the CV measurements were also performed in N_2 by switching to N_2 flow through the electrochemical cell. The CVs of the catalysts in a solution of 10 mM $Fe(CN)_6{}^{3-/4-}$/1.0 M KCl at the scan rate of 50 mV/s were also conducted for assessing their effective surface areas.

1.3.2 Rotating disk electrode (RDE) measurement

For the RDE measurements, catalyst inks were prepared by the same method as CV's. 10 μL ink (containing 40 μg catalyst) was loaded on a glassy carbon rotating disk electrode of 5 mm in diameter (Pine Instruments) giving a loading of 0.2 mg/cm^2. The working electrode was scanned cathodically at a rate of 5 mV/s with varying rotating speed from 400 rpm to 2025 rpm. Koutecky–Levich plots (J^{-1} vs. $\omega^{-1/2}$) were analyzed at various electrode potentials. The slopes of their best linear fit lines were used to calculate the number of electrons transferred (n) on the basis of the Koutecky-Levich equation:

$$\frac{1}{J}=\frac{1}{J_L}+\frac{1}{J_K}=\frac{1}{B\omega^{1/2}}+\frac{1}{J_K} \qquad \text{(S1)}$$

$$B=0.62\text{n}FC_0(D_0)^{2/3}\upsilon^{-1/6} \qquad J_K=\text{n}F\kappa C_0 \qquad \text{(S2)}$$

where J is the measured current density, J_K and J_L are the kinetic- and diffusion-limiting current densities, ω is the angular velocity, n is transferred electron number, F is the Faraday constant, Co is the bulk concentration of O_2, v is the kinematic viscosity of the electrolyte, and k is the electron-transfer rate constant.

For the Tafel plot, the kinetic current density measured at a rate of 5 mV/s with a rotating speed from 1600 rpm was calculated from the mass-transport correction of the RDE data by:[7]

$$J_K=\frac{J\times J_L}{(J_L-J)} \qquad \text{(S3)}$$

1.3.3 Rotating ring-disk electrode (RRDE) measurement

For the RRDE measurements, catalyst inks and electrodes were prepared by the same method as RDE's. The ink was dried slowly in air and the drying condition was adjusted by trial and error until a uniform catalyst distribution across the electrode surface was obtained. The disk electrode was scanned cathodically at a rate of 5 mV/s and the ring potential was constant at 1.3 V vs. RHE. The % HO_2^- and the electron transfer number (n) were determined by the followed equations[8]:

$$\%HO_2^-=200\times\frac{I_r/N}{|I_d|+I_r/N} \qquad \text{(S4)}$$

$$n=4\times\frac{|I_d|}{|I_d|+I_r/N} \qquad \text{(S5)}$$

where I_d is disk current, I_r is ring current, and N is current collection efficiency of the Pt ring. N was determined to be 0.40 from the reduction of $K_3Fe[CN]_6$.

1.3.4 Electrical conductivity measurement

Typically, the catalysts were mixed with 1.0 wt% polytetrafluoroethylene (PTFE, solid powder, Dupont) as a binder, and homogenized in an agate mortar. The mixture

was rolled into 80–100 μm thickness sheets, pressed by 20 MPa and finally cut into 3 × 1 cm^2 sheets. Then the obtained sheets were coated with silver conductive adhesives on both sides and tested using a multimeter. The conductivity of the catalysts was calculated using the formula:

$$\lambda = \frac{L}{R_x W d} \quad \text{(S6)}$$

where λ is the electrical conductivity of the catalyst, L, W, d is the length, width and thickness of the sheet, respectively, and R_x is the resistance of the catalysts tested by the multimeter.

1.3.5 XPS analysis

For the XPS data analysis, the Shirley background was subtracted before curve fitting. The experimental spectra were fit into components of Gaussian line shape. The elemental compositions were determined by the ratios of peak areas corrected with the sensitivity factors. For N 1s and C1s peaks of the CoO/NGHSs before and after etching, the line widths (fwhm) of the components with the same oxidation states were kept relative constant to minimize the fitting errors.

1.3.6 RHE calibration

The saturated calomel electrode (SCE) used as the reference electrode was calibrated with respect to reversible hydrogen electrode (RHE). The calibration was performed in a high-purity H_2 (99.999%) saturated electrolyte with two Pt wires as the working and counter electrode, respectively. Cyclic voltammograms (CVs) were acquired at a potential scan rate of 1 mV/s, and the average of the two potentials at which the current crossed zero was taken as the thermodynamic potential of the RHE. In 0.1 M KOH, E(RHE) = E(SCE) + 0.992 V, and in 1.0 M KOH, E(RHE) = E(SCE) + 1.051 V.

2 SEM images of the GO/PS spheres and the GHSs

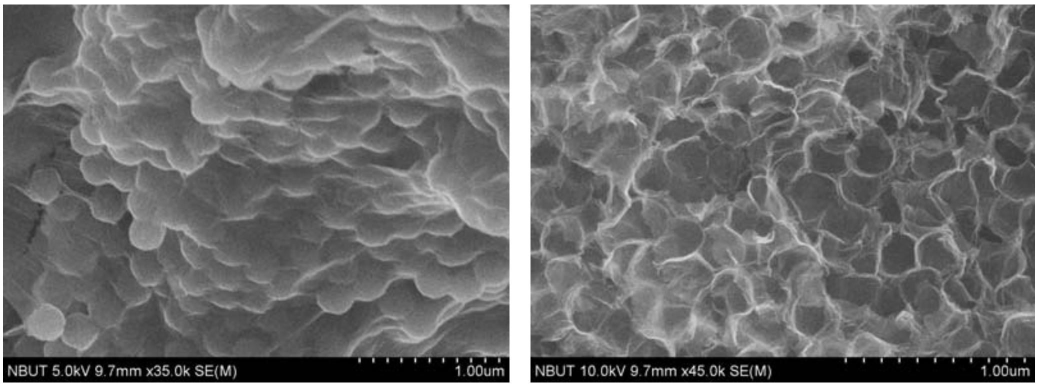

Figure S1. SEM images of (a) the GO/PS spheres and (b) the GHSs.

3 Higher-magnification and High resolution TEM image of the CoO/NGHSs

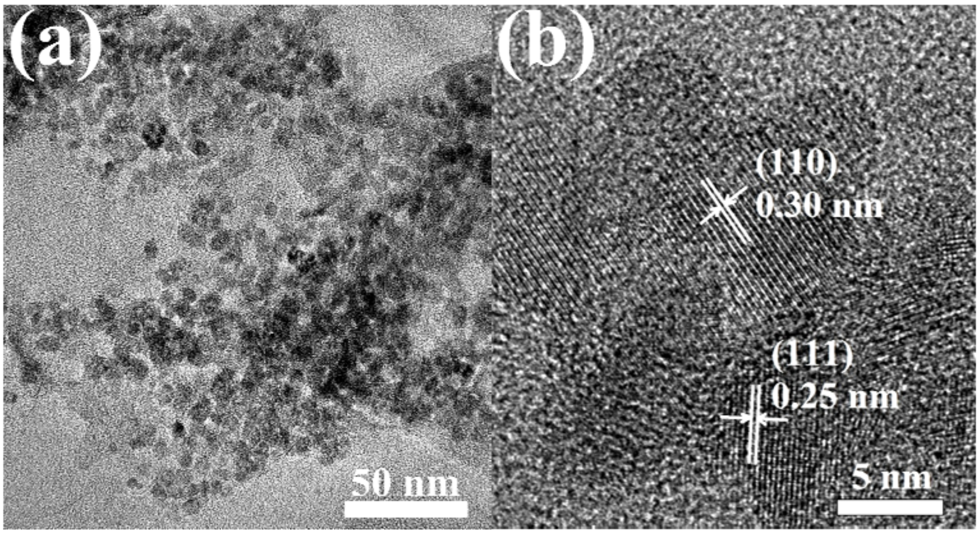

Figure S2. (a) Higher-magnification TEM image of the CoO/NGHSs. (b) High resolution TEM image of the CoO/NGHSs.

4 XPS survey spectra of the pure CoO solid and Deconvoluted Co2p of the CoO/NGHSs.

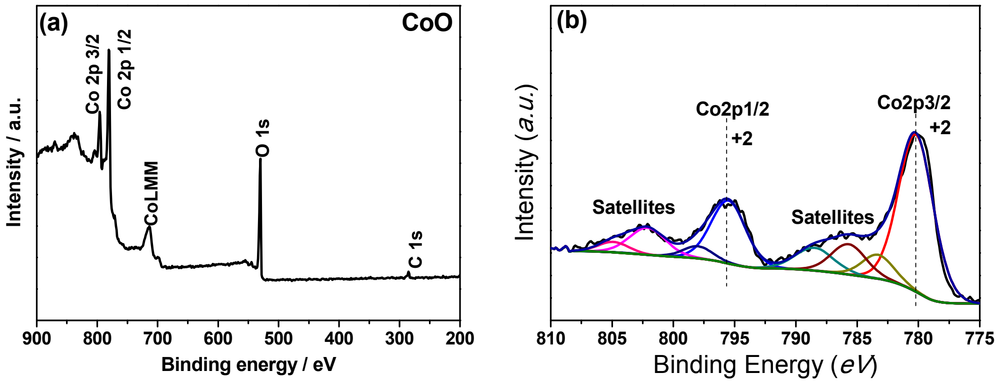

Figure S3. (a) XPS survey spectra of the pure CoO solid; (b) Deconvoluted Co2p of the CoO/NGHSs.

5 Calculation of the relative weight percentage of CoO in the CoO/GHSs and the CoO/NGHSs

5.1 The relative weight percentage of CoO in the CoO/GHSs: There are two methods that can be used to calculate the relative weight percentage of CoO in the CoO/GHSs. The both methods give the comparable values for the relative weight percentage of CoO in the CoO/GHSs.

Method I: Since the weight increase in the TGA curve of the CoO/GHSs (Figure S2) is caused by the oxidation of CoO to Co_2O_3. So, if we assume that the relative weight percentage of CoO in the CoO/GHSs is x%, then:

$$(100\% - x\%) + x\% \times \frac{0.5MW_{Co_2O_3}}{MW_{CoO}} = 105.3 \quad \text{(S7)}$$

Where $MW_{Co_2O_3}$ and MW_{CoO} are the molecular weight of Co_2O_3 (165.8) and CoO (74.9), respectively. The solution of Eq. S7 gives x=49.6. Therefore, the relative weight percentage of CoO in the CoO/GHSs is 49.6%

Method II: Since the remaining TGA product of the CoO/GHSs is Co_2O_3. Therefore,

$$x\% \times \frac{0.5MW_{Co_2O_3}}{MW_{CoO}} = 52.0\% \quad \text{(S8)}$$

The solution of Eq. S8 gives x=47.0. Therefore, the relative weight percentage of CoO in the CoO/GHSs is 47.0%, which is very close to the value obtained from ***Method I***.

5.2 The relative weight percentage of CoO in the CoO/NGHSs: Due to the interference of the weight increase at the temperature range from 130 to 340 ^{o}C by the weight loss due to the loss of the physically adsorbed water, we directly used the *Method II* to calculate the relative weight percentage (y%) of CoO in the CoO/NGHSs, i.e.:

$$y\% \times \frac{0.5MW_{Co_2O_3}}{MW_{CoO}} = 52.9\% \quad \text{(S9)}$$

The solution of Eq. S9 gives y=47.8. Therefore, the relative weight percentage of CoO in the CoO/NGHSs is 47.8%.

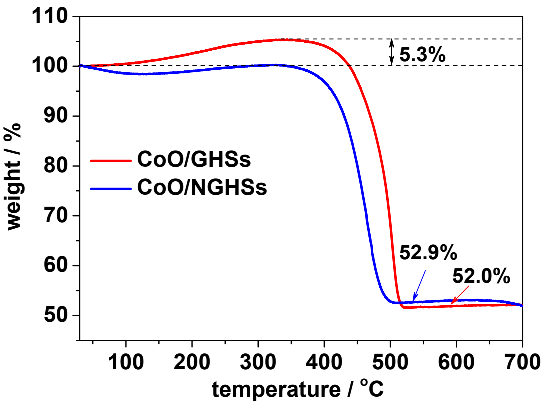

Figure S4. TGA curves of the CoO/GHSs and the CoO/NGHSs.

6 Electroactive surface areas of the catalysts

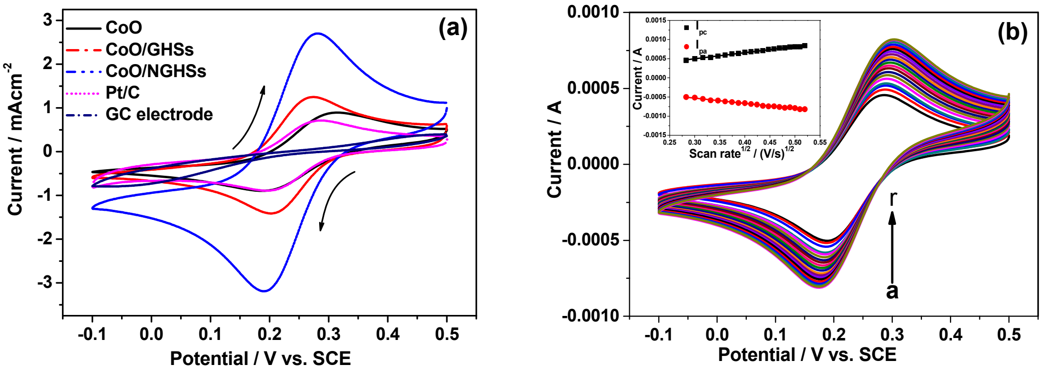

Figure S5. (a) CVs of the pure CoO solid, the CoO/GHSs, the CoO/NGHSs, and the Pt/C 20 wt. % casted on the glassy carbon (GC) electrode in 10.0 mM $Fe(CN)_6^{3-/4-}$ in 1.0 M KCl. The scan rate was 50 mV/s. For the comparison, the CV of the bare GC electrode was also measured. (b) CVs of the CoO/NGHSs on GC electrode in 10 mM $Fe(CN)_6^{3-/4-}$/1.0 M KCl at various scan rates from 80 mV/s to 270 mV/s. Inset: plot of peak current vs. $(\text{scan rate})^{1/2}$ of the CoO/NGHSs casted on the GC electrode. The loadings of the catalysts on GC electrode kept the same and were ~0.2 mg/cm^2 for all the cases.

The electrocatalytic surface areas of the catalysts were determined by cyclic voltammetry using 10 mM $Fe(CN)_6^{3-/4-}$ in 1.0 M KCl, as shown in Figure S3a. Figure S3b shows that the $\Delta E_p = (E_{pa} - E_{pc})$ of the CoO/NGHSs increases with increasing scan rate, but the formal potential ($E^{0'} = 1/2(E_{pc} + E_{pa})$) is almost constant, indicating the quasi-reversibility of the electron transfer process.[9] The electroactive surface area can then be estimated according to the Randles-Sevcik equation[10-11]:

$$i_p = 2.99\times10^5 nACD^{1/2}\upsilon^{1/2} \quad \text{(S10)}$$

$$A = k/(2.99\times10^5 nCD^{1/2}) \quad \text{(S11)}$$

where i_p, n, A, C, D, and υ are the peak current, the number of electrons involved in the reaction, the electroactive surface area, the concentration of the reactant, the diffusion coefficient of the reactant species, and the scan rate, respectively. k is the slope of the straight line for i_p *vs.* $\upsilon^{1/2}$. The redox reaction of $Fe(CN)_6^{3-/4-}$ involves one-electron transfer (n =1), and the diffusion coefficient (D) is 6.30×10^{-6} cm^2/s. Based on the results shown in Figure S3a, the electroactive surface areas of the pure CoO solid, the CoO/GHSs, the CoO/NGHSs, and the Pt/C 20 wt.% are 3.4×10^{-2}, 6.8×10^{-2}, 21.1×10^{-2}, and 2.53×10^{-2}, respectively. This indicates that the electroactive surface area of CoO/NGHSs is higher than the pure CoO solid, the CoO/GHSs, and the Pt/C 20 wt. %.

7 LSV curves of the catalysts for the ORR and corresponding K-L plots

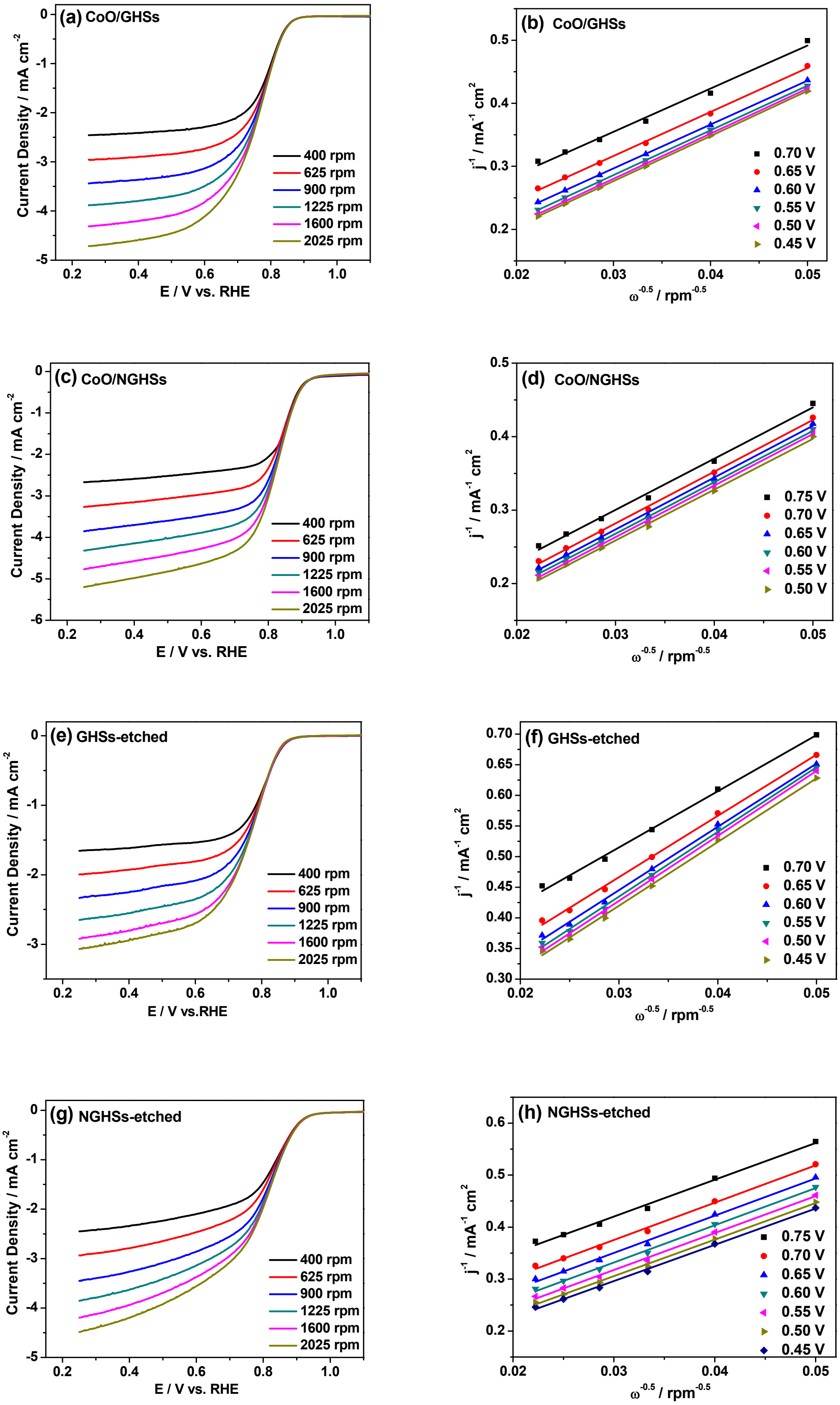

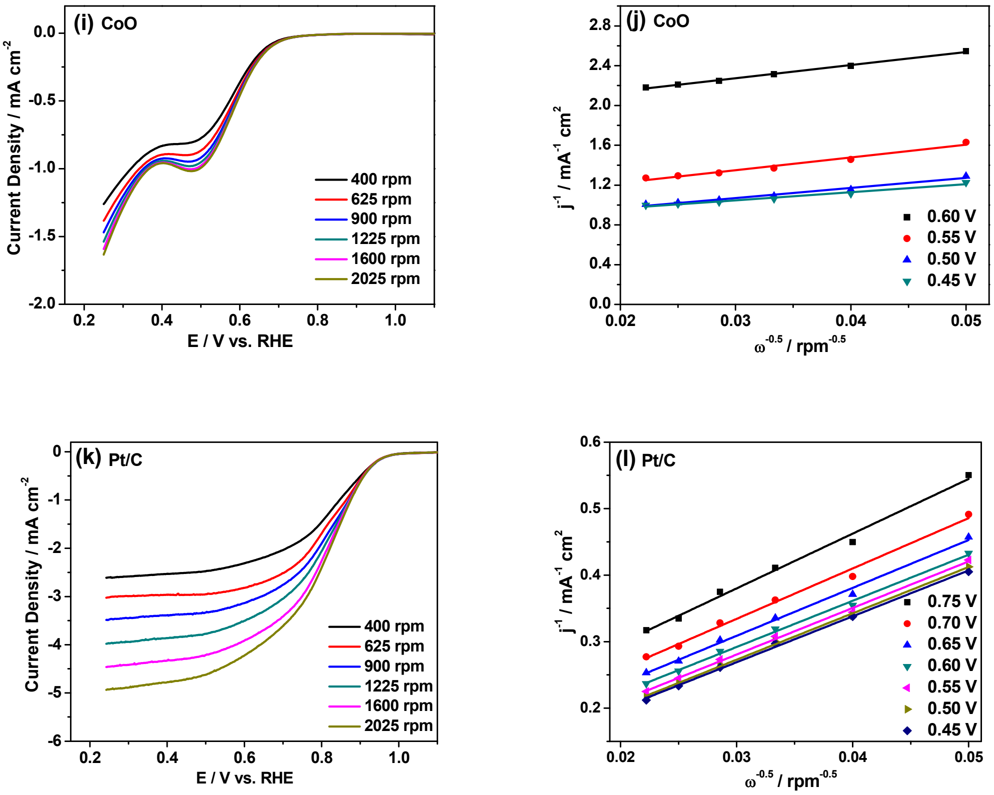

Figure S6. LSV curves at various different rotation rates for the ORR and corresponding K-L plots for the (a, b) CoO/GHSs, (c, d) the CoO/NGHSs, (e, f) the GHSs-etched, (g, h) the NGHSs-etched, (i, j) the pure CoO solid, and (k, l) the Pt/C 20 wt. % in the O_2-saturated 0.1 M KOH solution. The electron transfer numbers for the CoO/GHSs, the CoO/NGHSs, the GHSs-etched, the NGHSs-etched, the pure CoO solid, and the Pt/C 20 wt. % at 0.50 V vs. RHE, calculated based on the K-L plots and Equation S1 and S2, are 3.76, 3.95, 2.93, 3.50, 2.32, and 3.98, respectively, which are in good agreement with those obtained from the rotating ring-dick electrode voltammograms.

8 SEM and TEM images of the CoO/NGHSs and the CoO/GHSs after the ORR and OER

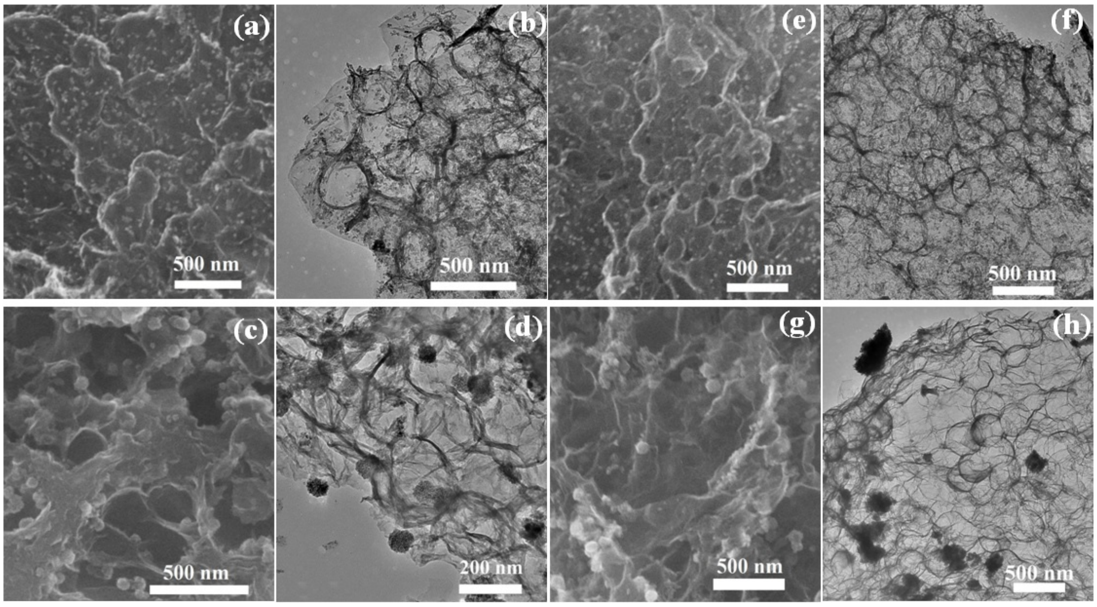

Figure S7. SEM and TEM images of (a, b) the CoO/NGHSs and (c,d) the CoO/GHSs after 10 h of the ORR at 0.75 V vs. RHE in an O_2-saturated 0.1 M KOH solution. SEM and TEM images of (e, f) the CoO/NGHSs and (g, h) the CoO/GHSs after 1500 cycles of the OER swept between 1.25 V and 1.75 V at 0.2 V/s in the N_2-saturated 1.0 M KOH electrolyte.

REFERENCES

1. Leng, W., Chen, M., Zhou, S.&Wu, L. Capillary force induced formation of monodisperse polystyrene/silica organic-inorganic hybrid hollow spheres. *Langmuir* **26** (17), 14271-14275 (2010).
2. Hidalgo-Alvarez, R., Moleon, J. A., De Las Nieves, F. J.&Bijsterbosch, B. H. Effect of anomalous surface conductance on ζ-potential determination of positively charged polystyrene microspheres. *J. Colloid Interf. Sci.* **149** (1), 23-26 (1992).
3. Hummers, W. S.&Offeman, R. E. Preparation of graphitic oxide. *J. Am. Chem. Soc.* **80** (6), 1339-1339 (1958).
4. Jiang, Z., Jiang, Z.-j., Tian, X.&Chen, W. Amine-functionalized holey graphene as a highly active metal-free catalyst for the oxygen reduction reaction. *J. Mater. Chem. A* **2**, 441-450 (2014).
5. Jiang, Z., Shi, Y., Jiang, Z.-J., Tian, X., Luo, L.&Chen, W. High performance of free-standing sulfonic acid functionalized holey graphene oxide paper as a proton conducting polymer electrolyte for air-breathing direct methanol fuel cells. *J. Mater. Chem. A* **2**, 6494–6503 (2014).
6. Jiang, Z.-J., Jiang, Z.&Chen, W. The role of holes in improving the performance of nitrogen-doped holey graphene as an active electrode material for supercapacitor and oxygen reduction Reaction . *J. Power Sources* **251**, 55-65 (2014).

7. Liang, Y., Wang, H., Zhou, J., Li, Y., Wang, J., Regier, T.&Dai, H. Covalent hybrid of spinel manganese–cobalt oxide and graphene as advanced oxygen reduction electrocatalysts. *J. Am. Chem. Soc.* **134** (7), 3517-3523 (2012).
8. Paulus, U. A., Schmidt, T. J., Gasteiger, H. A.&Behm, R. J. Oxygen reduction on a high-surface area Pt/Vulcan carbon catalyst: a thin-film rotating ring-disk electrode study. *J. Electroanal. Chem.* **495**, 134-145 (2001).
9. Zhang, G.&Yang, F. Electrocatalytic reduction of dioxygen at glassy carbon electrodes modified with polypyrrole/anthraquinonedisulphonate composite film in various pH solutions. *Electrochim. Acta* **52** (24), 6595-6603 (2007).
10. Guo, C. X., Lei, Y.&Li, C. M. Porphyrin functionalized graphene for sensitive electrochemical detection of ultratrace explosives. *Electroanal.* **23** (4), 885-893 (2011).
11. Guo, C., Hu, F., Li, C. M.&Shen, P. K. Direct electrochemistry of hemoglobin on carbonized titania nanotubes and its application in a sensitive reagentless hydrogen peroxide biosensor. *Biosens. Bioelectron.* **24** (4), 819-824 (2008).
